# Retraction Note: MIR600HG sponges miR-125a-5p to regulate glycometabolism and cisplatin resistance of oral squamous cell carcinoma cells via mediating RNF44

**DOI:** 10.1038/s41420-023-01711-8

**Published:** 2023-11-22

**Authors:** Xingguang Liu, Tengda Zhao, Zhe Yuan, Shaohua Ge

**Affiliations:** 1 Department of Oral and Maxillofacial Surgery, School and Hospital of Stomatology, Cheeloo College of Medicine, Shandong University & Shandong Key Laboratory of Oral Tissue Regeneration & Shandong Engineering Laboratory for Dental Materials and Oral Tissue Regeneration, Jinan, Shandong 250012 China; 2 Department of Oral and Maxillofacial surgery, Shandong Provincial Hospital Affiliated to Shandong First Medical University, Jinan, Shandong China; 3 The Affiliated Hospital of Stomatology, Guangdong Provincial Key Laboratory of Stomatology, Sun Yat-sen University, Guangzhou, Guangdong 510055 China

**Keywords:** Drug discovery, Cancer

Retraction to: *Cell Death Discovery* 10.1038/s41420-022-01000-w, published online 20 April 2022

The Editor in Chief has retracted this article. Concerns were raised about the apparent overlap between the two middle panels in Fig. 5H (si-RNF-44-1) and insufficient information about size of tumours in Fig. 7a. The authors did not provide raw data or proof that an ethics approval had been obtained before the beginning of the study. Additionally, Shaohua Ge stated they were not aware of the contents of the article, nor the submission process, because the email address provided on submission was not their email address. The authors did not state explicitly whether they agree to this retraction.

